# Web-Based Peer Navigation for Men with Prostate Cancer and Their Family Caregivers: A Pilot Feasibility Study

**DOI:** 10.3390/curroncol29060343

**Published:** 2022-06-15

**Authors:** Jacqueline L. Bender, Parminder K. Flora, Shimae Soheilipour, Mihaela Dirlea, Nandini Maharaj, Lisa Parvin, Andrew Matthew, Charles Catton, Leah Jamnicky, Philip Pollock, Winkle Kwan, Antonio Finelli, Arminée Kazanjian

**Affiliations:** 1 Cancer Rehabilitation and Survivorship, Department of Supportive Care, Princess Margaret Cancer Centre, Toronto, ON M5G 2C4, Canada; flora.parminder@gmail.com (P.K.F.); mihaela.dirlea@uhn.ca (M.D.); 2 Dalla Lana School of Public Health, University of Toronto, Toronto, ON M5T 3M7, Canada; 3 Institute of Health Policy, Management and Evaluation, University of Toronto, Toronto, ON M5T 3M7, Canada; 4 Division of Health Services and Policy, School of Population and Public Health, University of British Columbia, Vancouver, BC V6T 1Z3, Canada; shimae.soheilipour@dentistry.ubc.ca (S.S.); nandini.maharaj@ubc.ca (N.M.); lp327@georgetown.edu (L.P.); arminee.kazanjian@ubc.ca (A.K.); 5 Department of Oral Public Health, Torabinejad Dental Research Center, Isfahan University of Medical Sciences, Isfahan 8174673461, Iran; 6 Department of Surgery, Division of Urology, Princess Margaret Cancer Centre, Toronto, ON M5G 2M9, Canada; andrew.matthew@uhn.ca (A.M.); leah.jamnicky@uhn.ca (L.J.); antonio.finelli@uhn.capr (A.F.); 7 Department of Radiation Oncology, Division of Urology, Princess Margaret Cancer Centre, Toronto, ON M5G 2M9, Canada; charles.catton@rmp.uhn.ca; 8 Clinical Trials and Clinical Research, BC Cancer, Victoria, BC V8R 6V5, Canada; philip.pollock@bccancer.bc.ca; 9 Department of Radiation Oncology, BC Cancer, Fraser Valley, BC V3V 1Z2, Canada; wkwan@bccancer.bc.ca

**Keywords:** peer navigation, prostate cancer, digital health, family caregivers, supportive care

## Abstract

This study assessed the feasibility, acceptability and potential effects of True North Peer Navigation (PN)—a web-based peer navigation program for men with prostate cancer (PC) and their family caregivers. A one-arm, pre-post pilot feasibility study was conducted at two cancer centres in Canada. Participants were matched through a web-app with a specially trained peer navigator who assessed needs and barriers to care, provided support and encouraged a proactive approach to health for 3 months. Descriptive statistics were calculated, along with paired *t*-tests. True North PN was feasible, with 57.9% (84/145) recruitment, 84.5% (71/84) pre-questionnaire, 77.5% (55/71) app registration, 92.7% (51/55) match and 66.7% (34/51) post-questionnaire completion rates. Mean satisfaction with Peer Navigators was 8.4/10 (SD 2.15), mean program satisfaction was 6.8/10 (SD 2.9) and mean app usability was 60/100 (SD 14.8). At 3 months, mean ± SE patient/caregiver activation had improved by 11.5 ± 3.4 points (*p* = 0.002), patient quality of life by 1.1 ± 0.2 points (*p* < 0.0001), informational support by 0.4 ± 0.17 points (*p* = 0.03), practical support by 0.5 ± 0.25 points (*p* = 0.04) and less need for support related to fear of recurrence among patients by 0.4 ± 19 points (*p* = 0.03). The True North web-based peer navigation program is highly feasible and acceptable among PC patients and caregivers, and the associated improvements in patient and caregiver activation are promising. A randomized controlled trial is warranted to determine effectiveness.

## 1. Introduction

Prostate cancer (PC) is a highly prevalent condition [1]. In Canada, one in nine men will be diagnosed with PC during their lifetime [1]. While the age adjusted 5-year survival rate for prostate cancer is favourable at 93% [2], men face considerable anxiety when deciding on treatment options [3] and dealing with treatment side effects [4], which can impair physical and psychosocial functioning, and overall quality of life [5,6,7]. Common side effects of PC treatment include erectile dysfunction, urinary incontinence and bowel problems [5,6,7]. In addition, hormonal therapies can lead to body feminization, hot flashes, fatigue, loss of libido, osteoporosis and increase the risk of diabetes and cardiovascular disease [7]. 

Many men with PC lack access to relevant information and emotional support, and experience gaps in care when dealing with these difficult issues [8]. Lack of support from the healthcare system has been attributed to poor communication and continuity of care, and a lack of empathy on the part of healthcare professionals [9]. This is compounded by men’s general reluctance to seek help [10]. Unsurprisingly, several studies have documented a high burden of unmet needs in men with PC up to 10 years post-treatment, particularly in the areas of information and emotional support, psychological distress and sexual dysfunction [11,12,13,14,15]. In one population-based study, PC survivors had the highest level of unmet needs after treatment compared to any other cancer survivor group [16]. According to a systematic review, the most valued form of support men received following diagnosis of PC was one-to-one peer support [9]. However, many PC patients lack access to optimal peer support [16,17].

Family caregivers, who often coordinate care, assist with physical care and provide emotional support, have their own support needs [18]. For example, rates of anxiety and depression among cancer caregivers’ range from 10% to 55%, and in some cases exceed that of patients [18]. Family caregivers of men with PC face particular challenges [19]. They are often involved in the treatment decision, which can cause tension if priorities differ, and treatment side effects may affect their relationship with their partner [20,21]. A qualitative study found that PC family caregivers needed informational, emotional and practical support for themselves and effective medical care for the patient [20]. PC family caregivers’ needs were often unmet because of a lack of awareness of sources of help, lack of understanding of information, reluctance to ask for help, and prioritizing the patient’s needs [20].

Navigation could overcome gaps in access to care and support for PC patients and their family caregivers. Navigation in cancer care has been defined as the “proactive, intentional collaboration with a person and their family for the purpose of providing guidance as they negotiate the maze of treatment, services and potential barriers during the cancer journey” [22]. Navigators assess patients’ needs and, in collaboration with the patient and their family, develop a plan to overcome barriers to care while providing personalized support and in some cases care coordination. Several systematic reviews have shown that patient navigation improves screening rates and timeliness of diagnosis and treatment among cancer patients, and may also reduce healthcare costs [23,24,25,26]. Training navigators based on core competencies is critical for quality and effectiveness [24,25,27]. Navigation may be particularly valuable for men with PC, as well as, their family caregivers, given their reluctance to seek help [10,20]. However, there is limited research on navigation among male cancer patients and cancer family caregivers [25,28].

We developed True North Peer Navigation (PN) (https://peernavigation.truenth.ca/)—a web-based peer navigation program for men with PC and their family caregivers. In this program, PC patients or family caregivers are matched online with a specially trained peer navigator who assesses needs and barriers to care, provides practical, informational and emotional support, and empowers a proactive approach to managing health. The purpose of this study was to assess the feasibility, acceptability and potential effects of True North PN among men diagnosed with PC and their family caregivers.

## 2. Materials and Methods

### 2.1. Study Design

We conducted a pilot feasibility study of True North PN following the CONSORT reporting guidelines [29]. A single arm, pre-post, study design was employed [30]. As is recommended [31], the primary objective was to assess feasibility and acceptability of the program and the secondary objective was to assess potential effectiveness. The study was approved by the research ethics boards of the University Health Network and the University of British Columbia.

### 2.2. Setting

True North PN was implemented at cancer centres in Toronto and Vancouver, Canada. In Toronto, participants were recruited from the genitourinary oncology, sexual rehabilitation and androgen deprivation therapy clinics at the Princess Margaret Cancer Centre. In Vancouver, participants were recruited from the genitourinary oncology and radiation clinics at BC Cancer and the Prostate Cancer Supportive Care Program at the Vancouver Prostate Centre at the Vancouver General Hospital.

### 2.3. Study Participants

Participants were eligible to participate in the study if they were (a) diagnosed with localized, locally advanced or stable metastatic prostate cancer and receiving treatment or follow-up care at the participating cancer centres or were a family caregiver of a patient who met this criteria; (b) comfortable using computers and the Internet; and (c) able to read, speak and write in English. Patients were excluded if they had advanced metastatic disease or were palliative.

Participants were recruited during regularly scheduled clinic appointments. A member of their clinical team informed them about the study and gave them a study flyer. At the Princess Margaret Cancer Centre, interested individuals then met with a study coordinator in-person to learn about the study, assess their eligibility and if agreeable, provide their consent. At the BC Cancer Centre and Vancouver General Hospital, interested individuals were asked to provide written consent to be contacted by a research coordinator, who then explained the study, assessed their eligibility and sent them a consent form by email or postal mail.

### 2.4. Intervention

True North PN is an evidence-based, theory-informed, web-based peer navigation program that our team of health professionals designed and developed in collaboration with PC patients and caregivers, a healthcare design firm and a healthcare technology provider. It is based on a qualitative study that we conducted of the needs of PC patients and their family caregivers (9,17), as well as Stress and Coping Theory [32], Optimal Matching Theory [33] and Social Cognitive Theory [34]. It consists of (1) a web-based app co-designed and usability tested by patients/caregivers that includes a registration process, matching algorithm, participant and peer navigator profiles, private messaging, a health library of prostate cancer resources and a case management dashboard for peer navigators; (2) a 6-week peer navigator training program [35], that was shown to improve peer navigator knowledge and self-efficacy to perform core competencies [36]; and (3) peer navigator supervision and support. It is freely available for registered participants on the NexJ Connected Wellness platform (https://peernavigation.truenth.ca/).

Participants were provided a link to the True North PN app and instructed to: register an account; answer a series of questions to indicate their matching preferences (e.g., age, treatment, ethnicity, sexual orientation); view the profiles of available peer navigators who matched their criteria; select a navigator or be matched by the system administrator with an available navigator; communicate with their navigator through the app or by email, phone or in-person, and access resources from the health library on the program website for a duration of three months.

Navigators were instructed to contact patients/caregiver within 24 h of being matched through a personal message on the app (along with an email notification); conduct an assessment of the patient/caregivers’ needs and barriers to accessing care by telephone; establish a regular communication schedule (e.g., interactions every 2 weeks at minimum); provide support using the three-phase interaction guide and constellation of needs framework; and document their activities on the app using a standardized form that captured needs/barriers identified and activities performed for each interaction. Navigators were supervised by a clinical psychologist (AM) and the program leads (JLB and AM), who co-led monthly group debriefing sessions in-person or via video and provided one-on-one support. Real-time consultation and supervision of case management was conducted by the project team.

### 2.5. Outcomes

Feasibility assessment followed the CHERRIES reporting guidelines for web-based surveys [37] and the CONSORT reporting guidelines for eHealth interventions [38]. Specifically, feasibility was assessed in terms of recruitment rate (#consented/#approached), pre-intervention questionnaire completion rate (#completed pre-questionnaire/#consented), app registration rate (#registered on website/#completed pre-questionnaire), match rate (#matched with peer navigator/#registered on website) and post-intervention questionnaire completion rate (#completed post-questionnaire/#matched). 

Acceptability was assessed in terms of satisfaction, usability, usage and perceived benefits. Participant satisfaction with their navigator was assessed using the 9-item Patient Satisfaction with Navigator Interpersonal Relationship Scale (PSN-I) [39] (1 = strongly disagree to 10 = strongly agree). Program satisfaction was assessed using a 13-item program satisfaction measure that we developed for this program (1 = not at all satisfied to 10 completely satisfied; see Supplemental File S1). Usability of the app was assessed using the 10-item System Usability Scale (1 = strongly disagree to 5 = strongly agree) [40]. Usage of the app was assessed in terms of matching criteria completed by participants, interaction logs completed by peer navigators, messages exchanged between participants and peer navigators, and resources accessed from the health library by participants. For matching, participants were asked (1) if they wanted to be matched with a peer navigator who is similar to them based on specific characteristics (yes/no) and (2) the importance of seven matching criteria (incl., age, treatments received, sexual orientation, relationship status, ethnicity, spoken language and employment status) using a 4-point Likert scale (0 = not at all important to 4 = most important). Navigator interaction logs and messages exchanged on the app were used to determine the number of interactions per match and topics discussed, and resources accessed from the health library were used to determine the extent of health library use. Lastly, we assessed the perceived benefits of the program using a 20-item measure that we developed for the program based on patient-reported outcomes of a peer support telephone service [41] and feedback from patient partners (1 = strongly disagree to 10 = strongly agree; see Supplemental File S1).

Possible effects were explored using standardized patient and caregiver reported outcome measures. The Patient Activation Measure (PAM) [42] and Caregiver Activation Measure (CG-PAM) [43] were used to assess knowledge, skills and confidence to manage health related tasks. They are 13-item measures with 4-point Likert scales (strongly disagree to strongly agree) that produce scores from 0–100. The EQ-5D-5L [44] was used to assess health-related quality of life. It is a generic health utility instrument that consists of five domains of health status (mobility, self-care, usual activities, pain/discomfort and anxiety/depression) measured on five dimensions of severity. The Patient-Oriented Prostate Utility Scale (PORPUS) [45] was used to assess the PC-related quality of life of patients. It consists of ten domains (pain, energy, social support, communication with doctor, emotional well-being, urinary frequency and sexual function, sexual interest and bowel function) measured on four to six dimensions of severity. The Hospital Anxiety and Depression Scale (HADS) [46] was used to assess psychosocial well-being. It consists of two subscales designed to screen for anxiety (HADS-A) and depression (HADS-D) separately. Items are rated on a 4-point Likert scale (0 to 3), with some items reverse scored. The Enriched Social Support Inventory (ESSI) [47] was used to assess perceived social support. It consists of seven items, six of which are measured on a 5-point Likert scale and the seventh item, marital status is binary (yes/no). Lastly, the Supportive Care Needs Survey-Short Form (SCNS) [48] and the Supportive Care Needs Survey for Partners and Caregivers (SCNS-P&C) [49], were used to assess patient and caregiver supportive care needs, respectively. The SNCS is a 34-item measure rated on a 4-item scale (1 = no need, 2 = low need, 3 = moderate need and 4 = high need); the 8-item PC specific module was included. The SCNS-P&C is a 44-item measure that was developed based on the SCNS. We used the same 4-item response format that was used for the SCNS as it was determined by the scale developers to be more acceptable than its 5-item predecessor. 

Criteria for success were predetermined to be: >50% recruitment rate, >70% post-questionnaire recruitment rate, >80% peer navigator satisfaction, and improvements in patient and caregiver reported outcomes.

### 2.6. Analysis

Analysis was performed using SPSS version 21. Descriptive statistics were used to summarize feasibility, acceptability and outcome data. Secondary effectiveness outcomes were compared between groups using paired *t*-tests. Chi-square tests and *t*-tests were used to compare the sociodemographic and clinical characteristics of the participants who dropped out prior to matching (e.g., drop-outs) versus those who did not, as well as those who were matched and received support but did not complete the post-questionnaire (e.g., study non-completers) versus study completers. Patient and caregiver data were analyzed jointly and separately; findings are presented for the entire sample and for each group. Normality was assessed with graphical representations and Kolomogrov-Smirnov tests. All *p* values < 0.05 were considered significant

## 3. Results

### 3.1. Participant Characteristics

**Figure 1:**
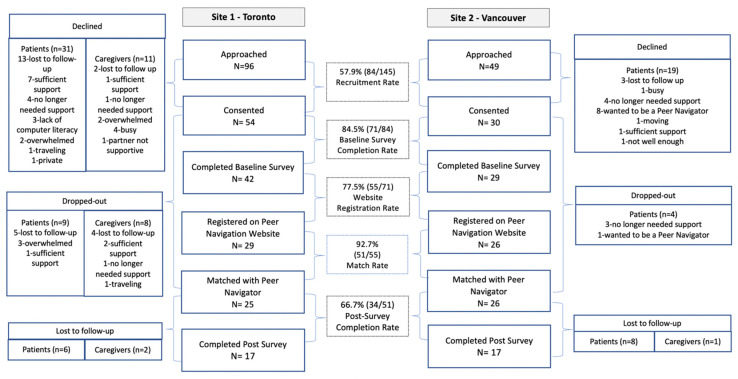
Participant flow diagram.

From November 2017 to April 2018, 145 participants were approached, 84 consented, 71 completed a pre-intervention questionnaire, 55 registered on the app, 51 were matched with a peer navigator (Figure 1). Of the 51 participants who were matched with a peer navigator, 43 were patients and eight were caregivers. Of the 34 participants who completed both pre- and post-questionaries, 29 were patients and five were caregivers. There were no significant differences in the sociodemographic and clinical characteristics between the participants who dropped out (*n* = 21) compared to those who received the intervention (*n* = 51), as well as between participants who did not complete post-questionnaire (*n* = 17) compared to study completers (*n* = 34).

The majority of participants were 65 years of age, White (*n* = 30, 88.2%), university educated (*n* = 21, 66.8%), heterosexual (*n* = 31, 91.2%) and in a relationship (*n* = 30, 88.2%) (Table 1). Most participants were diagnosed with localized disease (*n* = 30, 88.2%) and were either deciding on treatment (*n* = 8, 24.2%) or receiving follow-up care after treatment (*n* = 12, 36.4%). Of note, four of the 34 participants (2 patients and 2 caregivers) who completed the intervention were experiencing advancing disease.

### 3.2. Feasibility

The recruitment rate was 57.9% (84/145), pre-intervention questionnaire completion rate was 84.5% (71/84), app registration rate was 77.5% (55/71), patient/caregiver-peer navigator match rate was 92.7% (51/55) and post-intervention questionnaire completion rate was 66.7% (34/51) (Figure 1).

### 3.3. Acceptability

#### 3.3.1. Satisfaction

Participants’ satisfaction with their interpersonal relationship with their Peer Navigator was high (8.41/10, SD 2.5) (Table 2), satisfaction with the program was moderate (6.8/10, SD 2.9) (Table 3) and satisfaction with the usability of the app was moderate (60.0/100, SD = 14.8). The highest program satisfaction scores were reported for: the online matching process (7.4/10, SD = 3.2), app registration process (7.3/10, SD = 3.0) and support received from their navigator (7.0/10, SD = 3.7). Low satisfaction scores were reported for the health library (4.0/10, SD = 4.1) and the availability of program staff to answer programmatic (3.7/10, SD = 4.2) or technical (3.4/10, SD = 4.0) questions. Of note, caregivers reported lower program satisfaction scores compared to patients.

#### 3.3.2. Matching Preferences

Of the 49/51 participants who completed the matching process on the app, 71.4% (*n* = 35) wanted to be matched with a peer navigator who shared common characteristics, and 28.9% (*n* = 14) indicated that they would be fine being matched with any navigator. The most important matching criterion was spoken language (3.2/4, SD = 0.87) followed closely by treatments received (3.1/4, SD = 0.7), and then sexual orientation (2.5/4, SD = 1.1), current age (2.1/4, SD = 0.9), relationship status (1.9/4, SD = 1.1), ethnicity (1.3/4, SD = 1.1) and employment status (1.0/4, SD = 0.1).

#### 3.3.3. Participant–Peer Navigator Interactions

Navigators submitted 157 interaction notes on the app for interactions with participants conducted by phone or in-person: an average of 3.1 interaction notes per participant. The most common topics documented in the interaction notes were, in order of frequency: information (e.g., treatment and support options) (*n* = 40, 25%), state of mind (e.g., emotions, stress) (*n* = 35, 22%), side effects (e.g., incontinence, sexual functioning) (*n* = 27, 17%), other (*n* = 25, 16%), relationships (e.g., family, community) (*n* = 20, 13%), spirituality (e.g., personal growth) (*n* = 5, 3%) and practical concerns (e.g., finance, transportation) (*n* = 6, 4%). In addition, a total of 600 messages were exchanged on the app between the 51 matched participants and their peer navigators. Of the messages exchanged on the app, 342 messages were sent from Peer Navigators to participants (median 2, IQR = 6, range 1–44) and 258 messages (median = 1, IQR = 3, range 1–47) were sent from participants to Peer Navigators.

#### 3.3.4. Health Library Usage

The health library was accessed 22 times. Of the 51 participants who were matched with a navigator, 21.6% (*n* = 11) accessed at least one resource from the health library. 

#### 3.3.5. Perceived Benefits

The perceived benefits received from peer navigation were in the domains of validation support (7.8/10, SD = 2.4), followed by emotional support (7.6/10, SD = 2.6), autonomy support (7.5/10, SD = 2.5) and informational support (7.0/10, SD = 3.0) (Table 4). The top five item-level perceived benefits were feeling listened to (8.1/10, SD = 2.3), feeling that their thoughts and feelings were normal (8.1/10, SD = 2.1), feeling as though their peer navigator cared about them (8.0/10, SD 2.5), feeling more assured with their choice of treatment (7.8/10, SD = 2.3), feeling less anxious talking to a peer cancer survivor (7.7, SD = 2.4) and feeling more confident talking to their healthcare provider about their concerns (7.7, SD = 2.5). Of note, caregivers reported lower perceived benefit scores compared to patients.

### 3.4. Possible Effects

There were statistically significant improvements in mean pre/post-intervention scores for several outcome measures (Table 5). At 3 months, the largest difference was observed in patient and caregiver activation: mean ± SE patient and caregiver activation had increased by 11.5 ± 3.4 points. Other outcomes that improved included prostate cancer quality of life (Mpre = 97.1, Mpost = 98.2, *p* < 0.0001), informational support (Mpre = 3.5, Mpost = 3.9, *p* = 0.03), practical support (Mpre = 3.5, Mpost = 4.0, *p* = 0.04), less need for support related to fear of recurrence among patients (Mpre = 1.6, Mpost = 1.1, *p* = 0.03), greater met needs among caregivers (Mpre = 19, Mpost = 27, *p* = 0.05) and less need for support to look after oneself among caregivers (Mpre = 3.6, Mpost = 1.2, *p* = 0.02). In addition, there was a trend towards greater total met needs and fewer total unmet needs among patients and caregivers. No significant changes were recorded for ED5D-5L, or HADS anxiety or depression.

## 4. Discussion

To our knowledge, this is the first study of a web-based peer navigation program for people affected by cancer. This study demonstrates that the True North web-based peer navigation program is feasible to implement and evaluate among PC patients and family caregivers. Participants were highly satisfied with the support received from their peer navigator and reported several benefits. Further, participants experienced significant improvements in patient and caregiver activation, quality of life, social support and supportive care needs. These findings are promising and timely given the increased need for virtual supportive care interventions as a result of the COVID-19 pandemic [50].

The feasibility metrics met and, in some cases, exceeded our targets; they also revealed areas in need of improvement. We recruited and matched 51 participants within six months. These are promising feasibility outcomes. While peer support is highly valued by men with PC [9] and their family caregivers [20], access rates for both groups tend to be around 25–30% [51]. However, there was higher than expected drop-out prior to matching with a peer navigator and the post-questionnaire completion (67%), while acceptable, was also lower than expected. The main reason for not participating in the study was a lack of perceived need for support either because their need for support had passed or they felt they had adequate support. For men, the emasculating effects of treatment may have created additional barriers to help seeking along with a desire to normalize their experience by not seeking help [27,52]. In addition, research coordinators documented frustration with the number of steps involved in matching with a peer navigator and a lack of technology proficiency on the part of some participants. These findings indicate a need to streamline the registration and matching process, overcome barriers men may face in accepting support, offer the intervention at the time of greatest need, and incorporate additional strategies to increase follow-up response rates [53].

Patient navigation is fundamentally a relational process. Individuals typically judge navigators based on their empathy, warmth and caring demeanour more so than whether they are knowledgeable about every resource [39]. The Patient Satisfaction with Navigator Interpersonal Relationship Scale (PSN-I) was designed to measure these important dimensions of the navigator relationship. The mean PSN-I scores reported by participants in this study were high—8.4 for patients and 7.9 for caregivers—indicative of high satisfaction with navigators and comparable to other studies of navigated cancer patients [54]. Research has shown that individuals’ perceptions of the quality of their interpersonal relationship with their navigator impacts their satisfaction with their cancer care in general. Specifically, in a study of 1593 individuals who received navigation following abnormal cancer screening, patients navigated by higher rated navigators on the PSN-I scale reported higher satisfaction with their cancer care [54]. Using a measure of perceived navigational support that we developed for this study, we also demonstrated that participants gain multiple functional benefits from their relationships with their navigators. The most highly endorsed benefit reported in this study was in the domain of validation support, which is the type of social support peer relationships can provide about the appropriateness or commonality of thoughts, feelings and behaviours [55].

An important aspect of a person’s satisfaction with their interpersonal relationship with their navigator is the extent to which they feel that they can relate to their navigator [39]. This is the first known study to identify navigator matching characteristics. PC patients and caregivers wanted a peer navigator with whom they could speak in their preferred language, who had experience with the same treatments and treatment side effects that they were experiencing, and who was of the same sexual orientation. In contrast, racial or ethnic background was rated as the one of the least important matching criteria. Further research is warranted to investigate the importance of race and ethnicity as a navigator matching criterion as this sample consisted of predominantly white patients and caregivers. Racialized people face numerous health inequities and may also experience the disease differently due to cultural and religious beliefs [56,57]. 

Overall satisfaction with the program and perceived usability of the program website were lower than expected. Of note, caregivers reported lower program satisfaction scores on all items. It is not known why caregivers may have been less satisfied with the program or the app. However, it is worthy to note that two of the four caregivers were supporting partners with advancing disease and may have required more support than the program offered. The satisfaction item scores revealed that patient and caregiver participants were dissatisfied with the availability of program staff to answer their technical or program-related questions, and the resource library on the program website. Beyond recruiting, consenting and supporting the registration and matching process, program staff had minimal interaction with participants. Follow-up with participants revealed that some did not know that program staff were available to answer questions, or how to contact them if questions arose. We also learned that many participants found it difficult to find the resources in the library, while others had forgotten about resource library. They recommended following-up with participants after matching to check-in, answer any programmatic questions, and provide reminders about app features. In addition, we learned that the resource library’s search algorithm was performing sub-optimally and required improvements to yield more specific results.

Importantly, this study has demonstrated a significant association between peer navigation and improvements in patient and caregiver activation. No known prior studies have investigated the impact of navigation on patient or caregiver activation. Although an RCT of a one-on-one peer support program for men treatment for PC reported an increase in a self-efficacy, which is related to patient activation [58]. Patient and caregiver activation is defined as the knowledge, skills, beliefs and confidence to manage health related tasks [42]. It is a highly relevant outcome of navigation as more activated individuals are more likely to seek health care, adhere to treatment regimens and practice preventative care [59,60]. It also reflects PC patients’ and family caregivers’ expectations of peer navigation that we identified in our formative study (e.g., to be empowered to conduct health-related conversations, ask questions and proactively cope with treatment side effects) [17]. Further, there is evidence that individuals who are more activated, are more likely to engage in healthy behaviours and have clinical indicators that fall within the normal ranges compared to individuals who are less activated [60]. In this study, patient/caregiver activation rose by 11.4 points, corresponding to an increase from activation level three (taking action to maintain and improve one’s health) to activation level four (staying the course even under stress).

Additionally, we found an association between peer navigation and improvements in validated measures of patient quality of life, and patient/caregiver perceived social support and some supportive care needs; no significant differences were observed in measures of anxiety or depression. Similarly, prior randomized controlled trials of navigation for cancer, including among men with PC, have reported mixed effects on outcome measures of quality of life and no significant differences on measures of distress [24]. Of note, patient participants in this study reported significantly less need for support related to fear of recurrence, a type of cancer-related distress which might be a more relevant outcome of peer navigation [61,62]. Participation in this study was also associated with improvements in perceived informational and practical social support, but surprisingly not emotional support, as measured by the multidimensional enriched social support inventory. In contrast, a qualitative study of mainly female navigated cancer patients [63] reported that emotional support was a valued benefit of navigation, along with assistance with information needs, and problem solving. Qualitative research is warranted to explore PC patient and caregiver experiences with navigation further.

This study has certain limitations. This was a single arm feasibility study without a control group; a randomized controlled trial is warranted to determine effectiveness. The sample, although recruited from two provinces in Canada and an appropriate size for a feasibility study, was small, predominately White and university educated and may not reflect the experiences of those from diverse racial, ethnic or socioeconomic backgrounds. Likewise, as the study was conducted at two urban cancer centres with established survivorship programs, the findings may not be reflective of those located in rural or remote settings with less resourced hospitals. The sample of caregivers was small; hence, the caregiver findings should be interpreted with caution. While many of the caregiver outcomes were like those of patients, caregivers reported lower program satisfaction scores. Further research is needed to better understand the experience and impact of peer navigation among PC caregivers. The impact of the intervention on patient quality of life also requires further investigation as the baseline PORPUS measure was missing two items. It is also important to note that this study does not provide a complete picture of the total number of interactions between participants and peer navigators which was higher than reported as email interactions were not captured. In addition, the number of drop-outs and study non-completers is a limitation. While an assessment of the characteristics of drop-outs and study non-completers compared to completers found no differences, they may differ in other important ways. Lastly, the baseline levels of patient and caregiver activation were high (activation level three of four) reflective of an already, active and engaged sample. Future research should aim to reach less activated patients and caregivers who could potentially benefit more from navigation. 

## 5. Conclusions

A web-based peer navigation program is highly feasible and acceptable among PC patients and caregivers. The improvements in patient and caregiver activation are particularly promising. Addressing areas of improvement could enhance program satisfaction and effects. A randomized controlled trial is warranted to further investigate the effects of web-based peer navigation on patient and caregiver reported outcomes.

## Figures and Tables

**Table 1:** Participant characteristics.

Characteristic	Category	Count (%), Unless Otherwise Specified
Age (years): mean, (SD), range		65.2 (7.1), 48–78
Participant	Patient	29 (85.3)
	Caregiver	5 (14.7)
Sex	Male	29 (85.3)
	Female	5 (14.7)
Race/Ethnicity	Caucasian	30 (88.2)
	Asian	1 (2.9)
	Filipino	1 (2.9)
	South Asian	1 (2.9)
	West Indian	1 (2.9)
Relationship Status	Married/Common-Law/Dating	31 (91.2)
	Single/Widowed/Divorced	3 (8.8)
Sexual Orientation	Heterosexual	31 (91.2)
	Homosexual	2 (5.9)
	Prefer not to say	1 (2.9)
Education completed	University	21 (66.8)
	College	9 (26.8)
	Secondary school	4 (11.8)
Household Income	40,000 or less	2 (5.8)
	40,001–80,999	10 (29.4)
	80,001–100,000	5 (14.7)
	More than 100,001	13 (38.2)
	Prefer not to say	4 (11.8)
Type of disease	Localized	30 (88.2)
	Metastatic	3 (8.8)
	Not sure	1 (2.9)
Stage of Journey	Deciding on Treatment	8 (24.2)
	Active Surveillance	4 (12.1)
	In-treatment	5 (15.2)
	In- recovery/follow-up	12 (36.4)
	In-recurrence	4 (12.1)

**Table 2:** Satisfaction with interpersonal relationship with navigator (PSN-I).

Item		Mean (SD)	
	Total (*n* = 34)	Patient (*n* = 29)	Caregiver (*n* = 5)
1. Is courteous and respectful to me	8.9 (1.7)	8.9 (1.7)	8.8 (1.3)
2. Is easy to talk to	8.7 (2.0)	8.8 (1.9)	8.2 (2.5)
3. Makes me feel comfortable	8.6 (2.1)	8.6 (2.2)	8.4 (1.5)
4. Gives me enough time	8.5 (2.2)	8.6 (2.1)	7.8 (2.5)
5. Listens to my problems	8.5 (2.4)	8.6 (2.4)	8 (2.5)
6. Is dependable	8.3 (2.4)	8.4 (2.5)	8 (2.1)
7. Cares about me personally	8.2 (2.6)	8.3 (2.7)	8 (2.4)
8. Is easy for me to reach	8.2 (2.4)	8.3 (2.4)	8 (2.1)
9. Figures out important issues in my healthcare	7.8 (2.6)	7.9 (2.7)	7.2 (2.2)
Mean satisfaction	8.4 (2.5)	8.4 (2.2)	7.9 (2.1)

**Table 3:** Program satisfaction.

Item		Mean (SD)	
	Total (*n* = 34)	Patient (*n* = 29)	Caregiver (*n* = 5)
1. Online matching process	7.4 (3.2)	7.8 (3)	4.8 (3.3)
2. Process of registering on website	7.3 (3.0)	7.5 (3)	6.6 (3.6)
3. Support received from your navigator	7.0 (3.7)	7.3 (3.7)	5.8 (3.7)
4. Interactions with your navigator	6.8 (3.6)	7.3 (3.4)	3.6 (3.5)
5. Overall program satisfaction	6.8 (2.9)	7 (2.9)	5.6 (3.5)
6. Availability of your navigator	6.4 (3.9)	7 (3.8)	3.6 (3.5)
7. Interactions with your program staff	6.1 (3.7)	6.6 (3.6)	3.6 (3.6)
8. Support received from program staff	5.3 (4.2)	5.7 (4.3)	3.6 (4.2)
9. Length of the program	5.3 (3.9)	5.5 (4.1)	4 (2.9)
10. Messaging chat feature on website	5.3 (4.2)	5.8 (4.3)	2.6 (2.8)
11. Health library on website	4.0 (4.1)	4.1 (4.2)	3.6 (3.9)
12. Availability of program staff for program questions	3.7 (4.2)	4 (4.4)	2 (2.3)
13. Availability of program staff for technical questions	3.4 (4.0)	3.7 (4.3)	1.4 (1.5)

**Table 4:** Perceived benefits gained from interactions with navigator.

Item (*As a Result of My Interactions with My Navigator*…)		Mean (SD)	
	Total (*n* = 34)	Patient (*n* = 29)	Caregiver (*n* = 5)
*Informational Support*	*7.0 (3.03)*	*7.2 (3)*	*5.4 (3.5)*
1. I feel more informed about prostate cancer and its treatment	6.9 (3.1)	7.1 (3)	5.8 (3.8)
2. I feel more informed about resources and services	6.7 (3.1)	7 (3)	4.8 (3.5)
3. I feel more informed about the road ahead and what to expect	7.1 (3.1)	7.2 (3)	5.8 (3.8)
4. I have the information to move forward	7.2 (2.8)	7.5 (2.8)	5.3 (3)
*Emotional Support*	*7.6 (2.6)*	*7.8 (2.5)*	*6.2 (3.2)*
5. I feel less anxious	7.4 (2.8)	7.7 (2.6)	5.5 (3.7)
6. I feel less down or depressed	7.3 (2.7)	7.6 (2.5)	5.8 (3.8)
7. I feel less alone	7.6 (2.7)	7.9 (2.5)	5.8 (3.7)
8. I feel that my peer navigator cares about me	8.0 (2.5)	8.1 (2.5)	7.6 (2.3)
9. I feel more hopeful about the road ahead	7.4 (2.6)	7.6 (2.6)	6.3 (2.6)
*Validation Support*	*7.8 (2.4)*	*8 (2.3)*	*6.5 (2.6)*
10. I feel that my peer navigator and I have a similar understanding	7.6 (2.6)	7.8 (2.6)	6.5 (2.5)
11. I feel that my peer navigator understands me	7.8 (2.6)	7.9 (2.7)	6.8 (2.5)
12. I feel that my peer navigator listens to me and is interested in what I have to say	8.1 (2.3)	8.1 (2.3)	8 (2.4)
13. I feel less anxious talking to someone who has been in my shoes	7.7 (2.4)	8 (2.2)	5.3 (2.9)
14. I feel that my thoughts and feelings are normal	8.1 (2.1)	8.3 (1.8)	6 (2.9)
*Autonomy Support*	*7.5 (2.5)*	*7.7 (2.3)*	*6.1 (3.6)*
15. I have found new ways of looking at my situation	7.4 (2.4)	7.4 (2.2)	7 (3.8)
16. I feel more in control	7.4 (2.3)	7.4 (2.1)	7 (3.8)
17. I feel more assured and comfortable with my choice of treatment	7.8 (2.3)	7.9 (2.1)	6.8 (3.9)
18. I feel more confident that I can manage emotional distress	7.3 (2.7)	7.7 (2.4)	4.5 (3.4)
19. I feel more confident talking to my healthcare provider about my concerns	7.7 (2.5)	7.8 (2.4)	6.5 (3.7)
20. I feel more confident coping with my cancer	7.4 (2.6)	7.7 (2.3)	4.8 (3.1)

**Table 5:** Pre/post-intervention assessment of possible effects.

Variable		Pre-Test Mean (SD)	Post-Test Mean (SD)	Mean Difference (SE)	Paired *t*-Test	*p*-Value
Patient/Caregiver Activation (PAM-PT/CG),						
Patient (*n* = 28)		62.2 (20.93)	74.06 (16.45)	−11.86 (3.87)	−3.1	<0.01 *
Caregiver (*n* = 4)		51.38 (8.22)	59.3 (6.45)	−7.93 (1.65)	−4.79	0.02 *
Total (*n* = 34)		60.84 (20.03)	72.2 (16.2)	−11.37 (3.39)	−3.4	<0.01 *
Health Quality of Life (EQ5D-5L)						
Patient (*n* = 29)		0.85 (0.1)	0.87 (0.14)	−0.02 (0.02)	−0.78	0.45
Caregiver (*n* = 5)		0.87 (0.08)	0.87 (0.1)	−0.01 (0.06)	−0.1	0.93
Total (*n* = 34)		0.8 (0.1)	0.9 (0.1)	−0.1 (0.1)	−0.8	0.45
PC Quality of Life (PORPUS)						
Patient (*n* =28)		97.1 (1.39)	98.23 (0.99)	−1.13 (0.2)	−5.6	<0.01 *
Anxiety (HADS-Anxiety)						
Patient (*n* = 29)		4.97 (3.31)	4.66 (3.93)	0.31 (0.52)	0.6	0.55
Caregiver (*n* = 5)		9.6 (5.32)	9.4 (6.02)	0.2 (1.53)	0.13	0.9
Total (*n* = 34)		5.6 (3.9)	5.3 (4.5)	0.3 (0.48)	0.6	0.55
Depression (HADS-Depression)						
Patient (*n* = 29)		2.59 (2.31)	3.31 (3.5)	−0.72 (0.46)	−1.57	0.13
Caregiver (*n* = 5)		7 (3.94)	6.2 (5.22)	0.8 (1.83)	0.44	0.68
Total (*n* = 34)		3.2 (3.0)	3.7 (3.8)	−0.5 (0.47)	−1.1	0.29
Social Support (ESSI)						
Patient						
Total (*n* = 29)		28.35 (4.92)	29.24 (5.11)	−0.9 (0.88)	−1.02	0.32
Informational (*n* = 29)		3.62 (0.94)	4.07 (0.88)	−0.45 (0.19)	−2.37	0.03 *
Practical (*n* = 28)		3.93 (1.33)	4.21 (1.29)	−0.29 (0.26)	−1.11	0.28
Caregiver (*n* = 5)						
Total		19.8 (7.4)	23.2 (8.44)	−3.4 (1.57)	−2.17	0.1
Informational		2.8 (1.48)	2.8 (1.48)	0 (0.32)	0	1
Practical		1.2 (0.84)	3 (1.41)	−1.8 (0.49)	−3.67	0.02 *
Total, *n* = 34		27.1 (6.0)	28.3 (5.9)	−1.2 (0.79)	−1.6	0.12
Informational Support, *n* = 34		3.50 (1.0)	3.9 (1.1)	−0.4 (0.17)	−2.3	0.03 *
Practical Support, *n* = 34		3.51 (1.6)	4.0 (1.3)	−0.5 (0.25)	−2.1	0.045 *
Supportive Care Needs						
Patient	Total Met Needs, *n* = 28	30.0 (11.1)	31.9 (10.5)	−1.9 (2.4)	−0.8	0.45
	Total Unmet Needs, *n* = 28	11.7 (11.1)	9.9 (10.3)	1.8 (2.4)	0.7	0.47
	Fear of Recurrence Unmet Need, *n* = 28	1.5 (1.3)	1.1 (1.0)	0.4 (0.19)	2.3	0.03 *
Caregiver	Total Met Needs, *n* = 5	19.0 (10.9)	27.0 (13.5)	−8 (3.0)	−2.6	0.05 *
	Total Unmet Needs, *n* = 5	25.8 (10.8)	18.0 (13.5)	7.8 (3.0)	2.6	0.06
	Support to look after own health Unmet Need, *n* = 5	3.6 (0.5)	1.6 (1.3)	2. (0.55)	3.6	0.02 *

* *p* < 0.05 was considered significant.

## Data Availability

The data presented in this study are available on request from the corresponding author.

## Supplementary Materials

The following supporting information can be downloaded at: https://www.mdpi.com/article/10.3390/curroncol29060343/s1, File S1: Author-Developed Satisfaction and Benefits Measures.
