# Supplementary material for: Web-Based Peer Navigation for Men with Prostate Cancer and Their Family Caregivers: A Pilot Feasibility Study
Source: Curr Oncol. 2022 Jun 15;29(6):4285–99. doi: 10.3390/curroncol29060343 (PMC9221974; doi:10.3390/curroncol29060343)
Supplement: Supplementary file 1 [file curroncol-29-00343-s001.zip › curroncol-1722548-supplementary.pdf]

Supplementary File S1 – Author-Developed Satisfaction and Benefits Measures

Section A: Your Satisfaction with Program

A1. We would like to know about your views about the TrueNTH Peer Navigation Program.

1. Please use the scale below to rate your **level of satisfaction** with each the following aspects of the program:

Not at all Satisfied				Moderately Satisfied					Completely Satisfied
1	2	3	4	5	6	7	8	9	10
☐	☐	☐	☐	☐	☐	☐	☐	☐	☐

1) Overall satisfaction with the TrueNTH Peer Navigation Program______
2) The length of the program__________
3) Availability of your Peer Navigator to address your support needs_____
4) Your interactions with your Peer Navigator______
5) Support received from your Peer Navigator_____
6) Availability of program staff to address your program-related questions_______
7) Availability of program staff to address your technical-related questions_______
8) Your interactions with the program staff _____
9) Support received from the program staff_________
10) Process of registering on the program website_______
11) Approach to matching you with a Peer Navigator _____
12) Messaging/chat feature on the program website for communicating with your Peer Navigator______
13) Health library on the program website________

2. Perceived Benefits of Interactions with Your Peer Navigator

Now we would like to know more about any benefits you may have gained from your interactions with your Peer Navigator. Using the following scale, please indicate your level of agreement with the following statements.

As a result of my interactions with my Peer Navigator...

	1 Strongly Disagree	2	3	4	5	6	7	8	9	10 Strongly Agree
1. I feel more informed about prostate cancer and its treatment.	◇	◇	◇	◇	◇	◇	◇	◇	◇	◇
2. I feel more informed about resources and services.	◇	◇	◇	◇	◇	◇	◇	◇	◇	◇
3. I feel more informed about the road ahead and what to expect.	◇	◇	◇	◇	◇	◇	◇	◇	◇	◇
4. I have the information I need to move forward.	◇	◇	◇	◇	◇	◇	◇	◇	◇	◇
5. I feel less anxious.	◇	◇	◇	◇	◇	◇	◇	◇	◇	◇
6. I feel less down or depressed.	◇	◇	◇	◇	◇	◇	◇	◇	◇	◇
7. I feel less alone.	◇	◇	◇	◇	◇	◇	◇	◇	◇	◇
8. I feel that my Peer Navigator cares about me.	◇	◇	◇	◇	◇	◇	◇	◇	◇	◇
9. I feel more hopeful about the road ahead.	◇	◇	◇	◇	◇	◇	◇	◇	◇	◇
10. I feel that my Peer Navigator and I have a similar understanding about the nature of my concerns.	◇	◇	◇	◇	◇	◇	◇	◇	◇	◇
11. I feel that my Peer Navigator understands me and my situation.	◇	◇	◇	◇	◇	◇	◇	◇	◇	◇
12. I feel that my Peer Navigator listens to me and is interested in what I have to say.	◇	◇	◇	◇	◇	◇	◇	◇	◇	◇
13. I feel less anxious talking to someone who has been in my shoes.	◇	◇	◇	◇	◇	◇	◇	◇	◇	◇
14. I feel that my thoughts and feelings are normal and to be expected.	◇	◇	◇	◇	◇	◇	◇	◇	◇	◇
15. I have found new ways of looking at my situation.	◇	◇	◇	◇	◇	◇	◇	◇	◇	◇
16. I feel more in control.	◇	◇	◇	◇	◇	◇	◇	◇	◇	◇
17. I feel more assured and comfortable with my choice of treatment.	◇	◇	◇	◇	◇	◇	◇	◇	◇	◇
18. I feel more confident that I can keep the emotional distress caused by my cancer and its treatment from interfering with the things I want to do.	◇	◇	◇	◇	◇	◇	◇	◇	◇	◇

19. I feel more confident that I can tell my health care provider concerns I have even when he or she does not ask.	◇	◇	◇	◇	◇	◇	◇	◇	◇	◇
20. I feel more confident coping with my cancer or the effects of treatment on a day to day basis.	◇	◇	◇	◇	◇	◇	◇	◇	◇	◇
